# Double-Blinded Randomized Pilot Clinical Trial Comparing Cognitive Side Effects of Standard Ultra-Brief Right Unilateral ECT to 0.5 A Low Amplitude Seizure Therapy (LAP-ST)

**DOI:** 10.3390/brainsci10120979

**Published:** 2020-12-13

**Authors:** Nagy A. Youssef, William V. McCall, Dheeraj Ravilla, Laryssa McCloud, Peter B. Rosenquist

**Affiliations:** 1 Department of Psychiatry and Health Behavior, Medical College of Georgia at Augusta University, Augusta, GA 30912, USA; WMCCALL@augusta.edu (W.V.M.); LRAVILLA@augusta.edu (D.R.); LMCCLOUD@augusta.edu (L.M.); PROSENQUIST@augusta.edu (P.B.R.); 2 Eisenhower Army Medical Center, Department of Behavioral Health, Fort Gordon, GA 30905 USA

**Keywords:** electroconvulsive therapy, neurostimulation, seizure therapy, low amplitude seizure therapy, cognitive side effects, ECT, current titration, precision ECT, treatment-resistant depression, mood disorders

## Abstract

Background: Concerns over cognitive side effects (CSE) of electroconvulsive therapy (ECT) still limit its broader usage for treatment-resistant depression (TRD). The objectives of this study were to (1) examine the CSE of Low Amplitude Seizure Therapy (LAP-ST) at 0.5 A compared to Ultra-brief Right Unilateral (UB-RUL) ECT using Time to Reorientation (TRO) as the main acute primary outcome, and (2) to compare effects on depressive symptoms between the two treatment groups. Methods: Participants were referred for ECT, consented for the study, and were randomized to a course of LAP-ST or standard UB-RUL ECT. TRO and depression were measured by the Montgomery-Åsberg Depression Rating Scale (MADRS). Results: Eleven patients consented. Of these, eight with a current major depressive episode (MDE) of unipolar or bipolar disorders were randomized. TRO was faster for the LAP-ST (mean = 6.8 min; SE = 4.9) than standard RUL ECT (mean = 15.5 min; SE = 6.5). Depression improved similarly in the two arms of the study from baseline (MADRS: LAP-ST = 41.0; SE = 2.0, RUL = 39.0; SE = 3.8) to endpoint (MADRS score: LAP-ST = 8.0; SE7.2, RUL = 9.5; SE = 3.8). Conclusions: This pilot, randomized and blinded clinical trial, suggests that the LAP-ST (at 0.5 A) has faster reorientation and possibly lower CSE compared to standard RUL-UB ECT. Caution is advised in interpreting these results due to the small sample size of this pilot study. Thus, future studies with similar design are warranted for replicating these findings.

## 1. Introduction

Fifty to sixty percent of patients do not achieve a sufficient response after antidepressant treatment [1]. About a third of patients do not remit even after three or more trials of different standard therapeutics [2,3,4,5]. Thus, interventions that help treat treatment resistant depression (TRD) are an important priority [6]. The most effective treatment for TRD is Electroconvulsive therapy (ECT) [7,8,9,10]. ECT and medications can also be a helpful treatment for relapse prevention [10,11]. However, potential cognitive side effects (CSEs) of ECT are still of concern. Although cognition is a broad concept, in this study we examine time to reorientation (TRO) as an established best predictor to memory problems during and after a course of ECT [12,13,14]. That is to say, longer TRO predicts more likely memory problems.

In order to decrease CSEs of ECT, several modifications in treatment parameters and electrode placement have been done (spanning over five decades). These include the ultra-brief (instead of brief) pulse [15,16], use of rectangular wave (replacing sine wave) [17,18], development of right unilateral (RUL) electrode placement versus bilateral (BL) [19,20], and using the most effective electrical dosage (in term of electric charge) via stimulus titration for the individual seizure threshold [21] (instead of lack of individualized titration of charge). All these modifications have lowered the CSEs [22,23,24,25] of ECT.

However, even with these helpful modifications and advancements, CSEs still occur in some patients. CSE may also contribute to the stigma related to ECT [26,27]. This results in the underutilization of ECT [8,28], which can also have an important effect on the quality of life of patients with TRD and in improving their functionality [29,30,31].

In addition to the above modifications, a recent proof of concept (POC) non-randomized clinical trial suggested that a lower current amplitude may spare CSEs. In this POC, we described Low Amplitude Seizure Therapy (LAP-ST) [32]. The amplitude used in the POC trial was titrated with the current (if needed) from 0.5 A to 0.6 A. Thus, this pilot study examines LAP-ST in a randomized design with the current amplitude fixed at the lowest amplitude possible (0.5 A) to examine further the cognitive benefit/efficacy tradeoff. The standard current amplitude used in clinical practice of modern ECT is a fixed current of either 0.8 mA or 0.9 mA, depending on the device and local practices [33].

In theory, LAP-ST can be helpful because of the direct effect of lower electric current, but the principal reason is that LAP-ST has a more focal electric field (EF) [34,35]. The current amplitude is the primary factor that drives the EF into brain regions located deeper in the brain and is responsible for cognition and memory such as the hippocampus and temporal lobe [33,35]. This extra focality of the EF can minimize or avoid high EF stimulation to the deeper hippocampal and temporal lobe regions [33,35]. Minimization of deeper stimulation by using LAP-ST is hypothesized to minimize CSE of ECT. In theory, this should not affect the more superficial cortical regions implicated in the antidepressant efficacy including the Dorsolateral Prefrontal Cortex (DLPFC) [36].

The aims of this pilot randomized study were as follows: (1) Examine whether LAP-ST minimizes CSEs compared to Ultrabrief RUL (UB-RUL) ECT (the standard ECT with the least CSEs) using Time to Orientation (TRO). TRO was the primary outcome, as it is the most sensitive to change [12]; (2) Compare effect on depressive symptoms between the two treatment groups. This study was intended to be as a pilot study and not a definitive trial.

## 2. Methods

### 2.1. Study Design and Participants

This clinical trial was approved by the Medical College of Georgia’s (MCG) Institutional Review Board (IRB), and the study was registered in ClinicalTrials.gov Identifier: NCT02583490. Participants were recruited from patients referred to the clinical ECT program after providing informed consent.

The trial was double-blinded (in that both study participant and rater were blind to treatment assignment), and participants were randomly allocated to the standard UB-RUL ECT or the LAP-ST. Random allocation was performed using four-per-block randomization to minimize imbalance between the two treatment groups. While to our knowledge the study was fully blinded, we did not test the effectiveness of the blinding.

Participants had to fulfill the following inclusion criteria: (1) they were referred to ECT by their clinician in a major depressive episode (MDE) using (DSM-IV criteria, either as part of major depressive disorder or bipolar disorders), (2) were over 20 years old, (3) had a Montgomery-Åsberg Depression Rating Scale (MADRS) of equal to or greater than 20 [37], (4) were using an effective method for birth control (for females of child-bearing age), (5) were stable medically, with no expected need to change their psychiatric medications during the trial, and (6) were able to provide valid informed consent.

Potential participants were excluded if they had a medical or neurological condition that could affect safe delivery of ECT, skull defect, implanted devices that affect safe ECT delivery, substance abuse or dependence within 1 week of starting the study, marked cognitive impairment, or if they were females who planned pregnancy or who were pregnant or breast-feeding.

### 2.2. Assessments

Patients were diagnosed using DSM IV criteria (assisted by Structured Clinical Interview, SCID) [38]. The 14-question TRO test was used as the primary cognitive outcome measure [12]. Time to reorientation was assessed at baseline, 1–3 days before starting the LAP-ST course of ECT (course to ensure that the participants can answer it all correctly at baseline). Then TRO was done after every session (after the end of the seizure as shown by EEG). It was done again 1–3 days after the end of the ECT or LAP-ST course. TRO was scored by determining the duration of time to answer all 14 out of 14 questions correctly [12,32]. Mean of TRO was calculated by averaging the TRO for all sessions, except the first titration session to capture the treatment parameters of UB-RUL ECT and LAP-ST during the treatment course.

Depressive symptom and clinical condition change were measured using MADRS [37] before starting the course (baseline), and about one to two days after the endpoint, as well as in the morning of each session of standard UB-RUL ECT or LAP-ST.

### 2.3. Procedure

Sessions were administered three times/week in both treatment conditions. Before each session, medication and anesthesia were administered as per standard care, generally using approximately 1 mg/kg methohexital for induction (unless indicated otherwise clinically), and approximately 0.75 mg/kg succinylcholine for muscle relaxation. Patients were ventilated with O2 100% starting at the anesthetic induction to the return of spontaneous respirations.

EEG monitoring was performed by two EEG channels (fronto-mastoid electrodes) to monitor brain seizure activity and duration. Motor seizures were observed by the cuff method applied to the lower extremity. MECTA Spectrum 5000Q device (MECTA Corporation, Tualatin, OR, USA) and RUL electrode placement were used in both treatment conditions. Estimation of seizure threshold (ST) was performed using the empiric titration method during the first session. For seizure titration, parameters were fixed for 0.3 pulse width and 0.9 A for the Standard UB-RUL and 0.5 A in the LAP-ST. Titration then occurred by increasing the train duration, and then stimulus pulse frequency at the initial titration session. The subsequent treatments were then performed at six times of the determined ST at the initial session for both treatment conditions. Titration parameters for the first treatment and subsequent treatments were presented in a prior publication [39].

### 2.4. Data Analysis

Sample characteristics were reported as mean (standard error) for continuous variables and as percentages for categorical variables. Due to sample size limitations, rating scales were reported using descriptive statistics in this pilot study. This study was intended to be a pilot study and thus power calculation was not done a priori (and is not powered enough for definitive inferential statistics). The program used for analysis was SPSS version 23, (Inc., Chicago, IL, USA).

## 3. Results

### 3.1. Demographic and Clinical Characteristics

**Figure 1:**
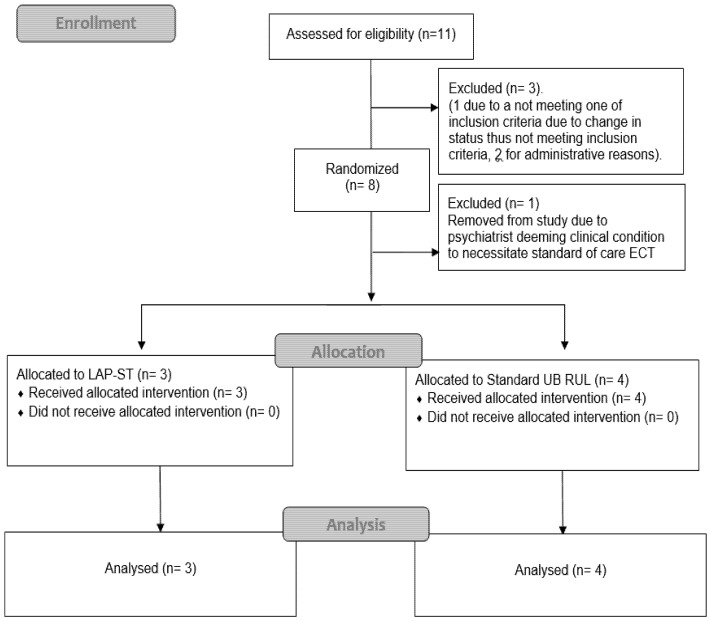
CONSORT Diagram: Flow diagram of the progress through the phases of the of two parallel groups randomized trial.

Eleven patients were enrollment in this pilot clinical trial. One patient was not included as his condition changed after consenting and did not meet an inclusion criterion, and two patients were not included for administrative reasons. After randomization, one patient’s treatment was discontinued by the ECT psychiatrist before the ECT treatment session on clinical grounds, as the treating psychiatrist deemed the condition to necessitate standard of care treatment and needed to unblind the intervention (see Figure 1 “Consort diagram”). Thus, only eight patients were finally randomized, and seven completed at least one treatment session (session in which ECT charge was given at therapeutic levels) and were included in the intent to treat (ITT) descriptive analysis. Due to the limited sample size, the final number of patients analyzed were *n* = 7. There is some clinical heterogeneity in this sample, and more patients are needed for a more robust statistical analysis of data. Demographic characteristics of the total sample of study participants were presented in Table 1.

### 3.2. Treatment Characteristics and Parameters

The titration algorithm UB-RUL ECT and LAP-St as well as the mean of the parameters of titration in initial and subsequent sessions, and seizure duration are all presented in a prior publication [39]. The titration was done in strict execution of the a priori protocol in the method. In brief, pulse width and amplitude were both fixed. Pulse width was fixed at 0.3 s, and amplitude was fixed at 0.9 A for the Standard UB-RUL and 0.5 A in the LAP-ST. Titration then occurred by increasing the train duration, and then when further increase was needed, it was done by increasing stimulus frequency at the initial titration session. The subsequent treatments were then performed at six times the determined ST at the initial session for both treatment conditions. The total charge between the standard UB-RUL ECT and the RUL LAP-ST were kept close in each arm of the study (for each step of initial titration and subsequent treatment session) to keep the dosage similar. We used 6-times seizure threshold in general in subsequent sessions after the titration session.

### 3.3. Cognitive Assessment

**Figure 2:**
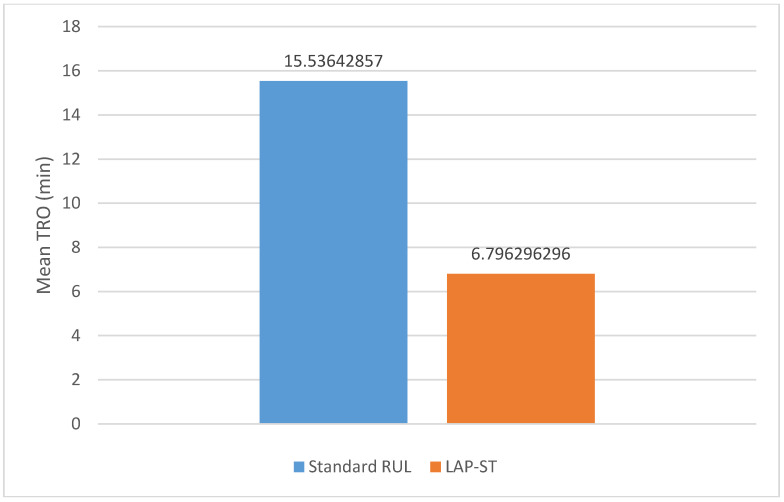
Time to Reorientation (TRO) for LAP-ST compared to UB-RUL-ECT: Mean TRO was much faster in the LAP-ST arm (6.8 min; SE = 4.9) than with standard UB-RUL ECT (15.5 min; SE = 6.5).

Mean TRO was much faster in the LAP-ST arm (6.8 min; SE = 4.9) than with standard UB-RUL ECT (15.5 min; SE = 6.5), predicting a more favorable cognitive effect for LAP-ST versus standard UB-RUL ECT. Graphic presentation of mean TRO is presented in Figure 2.

### 3.4. Efficacy Outcomes

**Figure 3:**
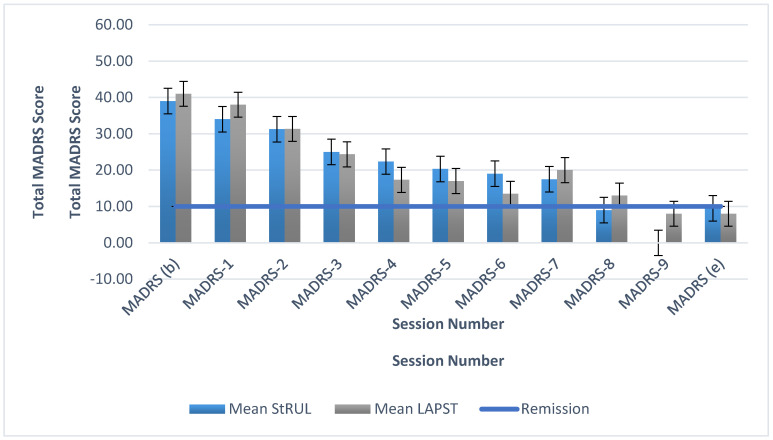
Means (SE) of MADRS scores for LAP-ST and UB-RUL ECT: Baseline MADRS score for LAP-ST = 41.0; SE = 2.0, and for RUL = 39.0; SE = 3.8. Endpoint MADRS score for LAP-ST = 8.0; SE = 7.2, and for RUL = 9.5; SE = 3.8.

Depression scores as measured by MADRS improved comparably (Figure 3) in both arms of the trial from baseline (MADRS: LAP-ST = 41.0; SE = 2.0, RUL = 39.0; SE = 3.8) to endpoint (MADRS score: LAP-ST = 8.0; SE = 7.2, RUL = 9.5; SE = 3.8). Given the small sample size, caution should be taken in interpreting the results.

## 4. Discussion

The present report is consistent with the initial proof of concept LAP-ST study (*n* = 22), providing further support for the premise that LAP-ST at 0.5 A has less CSEs (as measured by TRO) and preserved efficacy compared to standard UB-RUL ECT [32].

Longer TRO after ECT has been shown to prospectively predict long-term memory side effects [24,40,41]. Moreover, in prior studies TRO reported by using both standard RUL or bilateral ECT were much longer [9,24,25,42,43] than LAP-ST shown in this study.

This pilot study represents the first randomized and blinded clinical trial examining LAP-ST at 0.5 A compared to standard UB-RUL ECT. LAP-ST had numerically faster time to reorientation (Figure 2). The antidepressant efficacy was not different from the standard UB-RUL ECT group (Figure 3).

A recent larger randomized clinical trial [44] found less antidepressant effectiveness for 600 mA when comparing three different pulse amplitudes of 600, 700, and 800 mA. Although that may be the case, there are several differences between the two studies that are likely to explain difference in findings. For the purpose of future research directions for the field, we briefly outline some of these differences in the design between our study and the most recent low amplitude study [44]:

(1) There is an age difference between the two studies, with no restriction to 50 years and older in our study.
(2) Our study used TRO, rather than the Hopkins verbal learning test-revised retention raw score, as a primary outcome measure for cognition. TRO is commonly used as a sensitive cognitive outcome in the ECT and MST literature [12,13,14] that predicts memory side effects in the long term.
(3) Another difference is the use of three arms and dividing the stimulation to ultra-brief, and then brief pulse amplitude. This may have reduced the power of the study and the effectiveness of randomization (final of N of 3, 4, and 2 for brief-pulse at the end of the study for the 600, 700, and 800 mA arms respectively). Our study had two arms and had also a small sample size with three participants in one arm and four in the other with ultrabrief pulse. The brief pulse amplitude in the 3 arm study may have also been less efficient that the ultra-brief pulse [33]; as “briefer pulses require less total energy and charge to induce seizures than do longer pulses” [33].
(4) The current study was limited to all participants receiving RUL electrode placement, rather than allowing switching of some participants from RUL to BT within the study protocol.
(5) In this current study, we only followed the titration without allowing for change in parameters based on clinician’s adjustment, in order to decrease variability between the arms of the study as much as possible.
(6) The current study titration schedule allowed variability in frequency and train duration to simultaneously keep total dosage in terms of change similar and homogenous between the titration steps and treatment steps for the two arms of the study.

Thus, we recommend a future study with similar design to replicate the findings of this preliminary study before definitive conclusions. Future studies should examine amplitude range between 0.5 and 0.6 A. These levels of current amplitude are more likely to show difference in memory side effects (compared to standard of care current amplitude), while possibly further verifying efficacy. Future studies should have enough power, without minimizing power by having multiple subgroups at this stage of investigation. Current amplitudes below 0.5 A are unlikely to be effective in humans.

This current clinical trial used 14 questions for TRO, thus using more questions for orientation than other published trials. Achieving the rigorous cutoff of 14 correct questions [12] in this study was done in a mean of 6.8 min. This TRO was very similar to our prior open-label trial where TRO for LAP-ST of 6.6 min using also the 14-item TRO [12].

This study is limited by small sample size, and that elaborate cognitive batteries were not performed. TRO is, however, evidenced by several studies to be one of the most sensitive to change with the type of seizure therapy/ECT intervention, as well as most predictive of CSEs [24,40,41]. Prior data from continuation treatments for at least six months would suggest a continued benign profile or even improvement of CSEs from acute course to continuation treatment with more intervals between sessions [11,45].

Nonetheless, this pilot study has strengths, which include the double-blinded randomized design. We used in this study the most granular form of TRO, and thus arguably the most sensitive version of TRO [12]. The results of the current trial are promising, suggesting an antidepressant effect for both LAP-ST and standard RUL ECT, but LAP-ST may have an advantage of less CSEs. As mentioned, given the descriptive nature and clinical heterogeneity of this pilot trial of 0.5 A, and small sample size, the results are not definitive, and caution is advised. Thus, the results should be replicated in large clinical trials of LAP-ST at 0.5 A, with a longer follow-up period to show long-term effects of LAP-ST on cognition, depression, and suicidality [39]. This would add to the emerging literature [32,39,44] as a practical therapeutic alternative for TRD with less CSEs.

## Figures and Tables

**Table 1:** Demographic characteristics of participants.

Patient ID	Age	Gender	Race	Highest Education	Group	Included in the ITT Descriptive Analysis
1	55	Male	White	High School graduate	LAP-ST	Yes
2	56	Female	White	Some College	RUL	No
3	44	Male	Hispanic	High School graduate	RUL	No
4	31	Female	White	College	RUL	No
5	51	Female	White	College	RUL	Yes
6	29	Female	African American	College	RUL	No
7	27	Female	White	Some College	RUL	Yes
8	47	Male	African American	College	LAP-ST	Yes
9	59	Female	White	Master’s Degree	RUL	Yes
10	24	Male	White	Some College	LAP-ST	Yes
11	24	Male	White	Some College	RUL	Yes

RUL = Right Unilateral ECT; LAP-ST = Low Amplitude Seizure Therapy.
